# Resting-state EEG power and machine-learning classification in adult males with gambling disorder

**DOI:** 10.3389/fnhum.2025.1725528

**Published:** 2026-01-13

**Authors:** Metin Çınaroğlu, Eda Yılmazer, Selami Varol Ülker, Sultan Tarlacı

**Affiliations:** 1 Psychology Department, Faculty of Administrative and Social Science, Istanbul Nişantaşi University, Istanbul, Türkiye; 2 Psychology Department, Faculty of Social Science, Beykoz University, Istanbul, Türkiye; 3 Psychology Department, Faculty of Humanities and Social Science, Üsküdar University, Istanbul, Türkiye; 4 Medical School, Üsküdar University, Istanbul, Türkiye

**Keywords:** beta power, delta power, gambling disorder, machine learning, quantitative EEG, resting-state EEG

## Abstract

**Background:**

Gambling disorder (GD) is a behavioral addiction sharing neurobiological features with substance use disorders, yet objective biomarkers remain limited. This study examined resting-state EEG power and applied machine learning to identify potential electrophysiological markers of GD.

**Methods:**

Resting eyes-closed Electroencephalography (EEG) was recorded from 47 individuals with GD and 32 healthy controls. Absolute and relative power across delta (1–4 Hz), theta (4–8 Hz), alpha (8–13 Hz), and beta (13–30 Hz) bands were quantified over eight cortical regions. Group differences and correlations with the South Oaks Gambling Screen (SOGS) were analyzed. Multiple comparisons were controlled using the Benjamini–Hochberg False Discovery Rate (FDR) correction. A Linear Discriminant Analysis (LDA) classifier was trained to differentiate GD from controls based on EEG features.

**Results:**

Group differences in EEG power were subtle, with GD showing significantly higher delta power in the left temporal region (*p* = 0.032, *d* = 0.43). Within the GD group, greater gambling severity was associated with higher absolute beta power across frontal, parietal, temporal, and occipital regions (*r* ≈ 0.40–0.50, *p* < 0.01), and these associations remained significant after FDR correction (*p*FDR < 0.05). The LDA model using absolute power achieved 73.7% classification accuracy (AUC = 0.74), whereas relative power yielded near-chance accuracy (57.9%).

**Conclusions:**

GD is characterized by subtle but meaningful EEG alterations, particularly increased beta activity linked to gambling severity. Multivariate EEG patterns can distinguish GD from controls, supporting the potential of resting-state EEG as a biomarker for clinical assessment and severity monitoring in behavioral addiction.

## Introduction

Gambling disorder (GD) is increasingly recognized as a significant behavioral addiction with substantial public-health and societal consequences, yet its underlying neurobiological mechanisms remain incompletely understood (Abbott, 2020; Balodis and Potenza, 2020). Despite clinical overlap with substance-use disorders, GD still lacks reliable neurophysiological biomarkers for diagnosis, severity assessment, or treatment monitoring—a gap highlighted in recent work (Bel-Bahar et al., 2022; Simkute et al., 2024). Electroencephalography (EEG) has emerged as a promising tool for identifying such biomarkers because of its sensitivity to neural dynamics associated with arousal, executive functioning, and reward processing. Nevertheless, findings on resting-state EEG in GD remain inconsistent, underscoring the need for systematic investigation of spectral features that may characterize the disorder.

GD is recognized as a behavioral addiction that shares clinical and neurobiological features with substance-use disorders (Balodis and Potenza, 2020). Prior EEG studies have reported various alterations in individuals with GD, including subtle abnormalities in baseline spectral dynamics, hemispheric activity, and frontotemporal coherence, suggesting possible dysfunction in fronto-limbic circuits involved in stress and impulse control (Lee et al., 2017; Kim et al., 2018). Pathological gamblers also appear more likely to show dysfunctional EEG findings overall (Balconi and Angioletti, 2022); for instance, Regard et al. (2003) reported that approximately 65% of pathological gamblers demonstrated abnormal EEG activity—typically irregular temporal slow waves—compared to roughly 26% of healthy controls. Complementing these observations, more recent work has documented abnormalities in alpha rhythm organization and disturbances in slow-wave generation in GD, pointing toward broader disruptions in cortical oscillatory regulation (Rabadanova and Taygibova, 2020). Together, this literature suggests that EEG may capture meaningful neurophysiological deviations in GD, although the nature and consistency of these abnormalities remain unresolved.

Quantitative EEG studies further suggest that GD may be associated with deviations in power across specific frequency bands (Kim et al., 2019). For example, there is evidence that individuals with gambling or gaming addictions show elevated slow-frequency (delta and theta) power alongside reduced fast-frequency (beta) power at rest (Park et al., 2017). In clinical terms, increased delta/theta activity might reflect a state of cortical hypoarousal or impaired executive control (Harmony, 2013), whereas lower beta activity has been linked to inattention and impulsivity—a core feature of GD (Chowdhury et al., 2017). Notably, reduced beta power has been proposed as a candidate neurophysiological biomarker common to addictive disorders, correlating with diminished inhibitory control (Balconi et al., 2024). However, findings have not been entirely consistent across studies. Some investigations have failed to find robust group differences in resting EEG power between gamblers and non-gamblers, or have found that EEG differences emerge mainly when considering subgroups of GD (e.g., by trait impulsivity or other clinical features; Zilberman et al., 2018; Giustiniani et al., 2024; Dymond et al., 2014). These mixed results underscore the need for further research with larger samples and comprehensive EEG analyses to clarify baseline neural characteristics of GD.

Another open question is the clinical significance of any EEG differences in GD (Dubuson et al., 2023). If present, do neuroelectric alterations relate to the severity of gambling behavior? Prior studies hint that they might. Kim et al. (2018) observed that within a GD cohort, higher gambling severity was positively correlated with greater beta-band power in frontal and central regions. The authors suggested this heightened beta activity could indicate cortical hyperexcitability and increased craving in more severe gamblers. Similarly, recent work on GD patients stratified by impulsivity found systematic EEG variations: those with higher impulsivity had lower frontocentral theta, alpha, and beta power compared to low-impulsivity gamblers, implying that resting EEG measures are sensitive to individual differences in GD phenotype (Kamarajan et al., 2015). Taken together, these findings raise the possibility that EEG metrics might serve as objective indices of GD severity or subtypes, which would be of considerable clinical utility in assessment and treatment monitoring.

In light of the above, the present study set out to rigorously characterize resting-state EEG power in individuals with GD vs. healthy controls. We quantified absolute power in canonical frequency bands (delta, theta, alpha, beta) across multiple cortical regions, as well as relative power (the proportion of total EEG power each band represents) to capture any shifts in the spectral composition of brain activity. Based on prior literature, we hypothesized that the GD group would show increased slow-wave (delta/theta) power and reduced fast-wave (beta) power relative to controls, reflecting neural signatures of addiction (e.g., cortical underarousal and impulsivity-related dysregulation; Choi et al., 2013; Schutter and Kenemans, 2022). We further expected that within the GD group, EEG power measures would correlate with gambling severity—for instance, higher beta power might be associated with more severe gambling problems, consistent with earlier reports (Tabri et al., 2022; Navas et al., 2017).

Moreover, considering that subtle multivariate patterns in brain activity might distinguish GD patients even when univariate differences are small, we incorporated a machine learning approach (Dwyer et al., 2018). LDA was employed to test whether a combination of EEG features could reliably classify individuals as GD vs. healthy control (Tharwat et al., 2017). Machine learning has the advantage of detecting complex, distributed signal differences that may elude conventional statistical tests. In psychiatric research, EEG-based machine learning has been shown to identify subtle neural patterns associated with disorders that are not apparent on individual measures. We posited that an LDA model would achieve above-chance accuracy in separating gamblers from non-gamblers, thus demonstrating a distinctive multivariate EEG “fingerprint” of GD. In summary, our study aimed to bridge a gap in the GD literature by (1) comprehensively comparing resting-state EEG power spectra between GD and healthy individuals; (2) examining EEG correlates of clinical severity; and (3) exploring a data-driven classifier for GD based on EEG features. By doing so, we sought to deepen the clinical understanding of GD's neurophysiology and evaluate the potential of EEG as a tool for identifying and characterizing this disorder.

## Method

### Participants

A total of 79 adults participated in the study, including 47 individuals meeting DSM-5 criteria for GD and 32 healthy control (HC) subjects. All GD participants were recruited from outpatient addiction clinics in NP Hospital (Neuro Psychiatry full branch Brain Hospital affiliated by Üsküdar University) and had a history of pathological gambling behavior, while HCs were recruited from the same hospital who comes for regular checkups or for other reasons and had no psychiatric or neurological disorders. Because the accessible clinical population consisted almost entirely of male gamblers and the number of eligible female cases was too small for reliable analysis, the study sample was intentionally restricted to adult males. Accordingly, the findings of this study apply specifically to adult male individuals with gambling disorder. Groups were demographically matched; in particular, there was no significant difference in age between GD (mean = 32.5 ± 8.8 years) and HC (32.6 ± 9.9 years) participants (*t*(77) = 0.06, *p* = 0.95). All participants provided written informed consent in accordance with institutional ethical guidelines. Clinical severity in the GD group was assessed with the South Oaks Gambling Screen (SOGS), with all GD subjects scoring ≥5 (mean score in the moderate-to-severe range), whereas HCs scored below the pathological threshold (most scoring 0–1). Importantly, none of the GD participants— including those with mild psychiatric comorbidities—were using any psychotropic or neurologically active medications during the study period, ensuring that EEG recordings were not influenced by pharmacological effects. In addition to basic demographics, detailed psychosocial information was collected for all individuals with GD, including marital status, occupation, education level, and number of children. The GD sample included both married and single participants, with occupations spanning technical trades (e.g., technician, machinist), service-sector work, self-employment, and professional roles (e.g., teaching, law, architecture). Educational levels ranged from primary school to university and postgraduate degrees. Most participants were employed and socially integrated (e.g., married and/or with children), and none displayed psychosocial profiles suggesting extreme socioeconomic disadvantage or social isolation. Importantly, no psychosocial variable showed a pattern that would systematically influence resting-state EEG activity or bias group comparisons.

### South Oaks Gambling Screen—Turkish version (SOGS)

The South Oaks Gambling Screen (SOGS) was originally developed by Lesieur and Blume (1987) as a 20-item self-report measure to identify pathological gambling. The Turkish adaptation was carried out by Duvarci and Varan (2001) in two separate validation studies. During adaptation, three original items that did not discriminate pathological gamblers from controls in the Turkish context were removed and replaced with two culturally relevant items (e.g., borrowing from friends or converting gold/jewelry to cash). The final Turkish form consists of 19 scored items, with a cut-off score of 8 points yielding optimal sensitivity and specificity (both ≈90%) for identifying probable pathological gamblers according to DSM-IV criteria. Internal consistency was high (Cronbach's α = 0.88) and test–retest reliability over 1 month was excellent (*r* = 0.95). Scores range from 0 to 19, with higher scores indicating greater gambling-related problems; individuals scoring 8 or more are classified as probable pathological gamblers.

### EEG data acquisition and preprocessing

Resting-state EEG recordings were obtained from each participant in a quiet, electrically shielded room under dim lighting. EEG was recorded using a 19-channel active electrode system (ActiCap, Brain Products GmbH) following the international 10–20 system (Fp1/2, F3/4, F7/8, T3/4, T5/6, C3/4, P3/4, O1/2, Fz, Cz, Pz). Signals were acquired through NeuroGuide 2.5 software, referenced to linked mastoids (A1–A2) with a ground at Fpz. Electrode impedances were kept below 5 kΩ for all channels. Data were sampled at 250 Hz and filtered online with a 0.15–70 Hz band-pass filter and a 50 Hz notch filter to minimize electrical interference. Participants were instructed to sit comfortably and relax with their eyes closed during a 5-min resting-state recording. Preprocessing was performed using the Brainstorm toolbox (https://neuroimage.usc.edu/brainstorm). Raw EEG was visually inspected, and segments containing muscle or movement artifacts were manually rejected. When necessary, semi-automatic procedures (e.g., ICA-based ocular artifact removal) were applied. To ensure comparability across participants, a continuous, artifact-free 3-min epoch was extracted for spectral analysis. The cleaned EEG was re-referenced to the common average to reduce reference-related bias. Channels exhibiting persistent noise or poor signal quality were removed and replaced using spherical spline interpolation based on neighboring electrodes. To focus on canonical EEG rhythms, data were digitally filtered with a 0.5–30 Hz band-pass filter prior to frequency-domain analysis.

### Power spectral analysis and feature extraction

Processed EEG time series were subjected to Fast Fourier Transform (FFT) spectral analysis in Brainstorm to compute band-specific power measures. Power spectral density (PSD) was estimated for each electrode using Welch's method with 1-s Hamming windows and 50% overlap. Absolute power was calculated as the integral of the PSD within conventional frequency bands: delta (1–4 Hz), theta (4–8 Hz), alpha (8–13 Hz), and beta (13–30 Hz). For each participant, absolute power values were averaged within each band for predefined regions of interest (ROIs). ROIs were constructed by pooling electrodes over four bilateral cortical regions: left frontal (Fp1, F3, F7), right frontal (Fp2, F4, F8), left temporal (T3, T5), right temporal (T4, T6), left parietal (P3), right parietal (P4), left occipital (O1), and right occipital (O2). Midline electrodes (Fz, Cz, Pz) were excluded to maintain hemispheric specificity. Absolute power for each band and ROI was expressed in μV^2^.

Relative power was computed to index the proportion of total spectral energy contributed by each band in a given region. For each ROI and frequency band, relative power was calculated as:


$$\begin{array}{l} \text{Relative Power}=\frac{\text{Absolute Band Power}}{{\text{Total Power}}_{\left(\delta +\theta +\alpha +\beta \right)}}\times 100. \end{array}$$

Both absolute and relative power values were log-transformed when necessary to correct skewness and then *z*-score standardized across participants for further statistical analysis. In total, 32 absolute power features (4 bands × 8 ROIs) and 32 relative power features were derived per participant, yielding 64 spectral EEG features used in group comparisons and machine-learning classification.

### Multiple comparison correction (Benjamini–Hochberg FDR)

Given the large number of EEG–behavior correlations, we additionally applied the Benjamini–Hochberg False Discovery Rate (FDR) correction (Benjamini and Hochberg, 1995) to control for Type I error inflation. Compared to conservative procedures such as Bonferroni, FDR provides greater statistical power while maintaining the expected proportion of false discoveries below a chosen threshold (typically *q* = 0.05; Lix and Sajobi, 2010). Adjusted *p*-values (denoted as *p*FDR) were calculated using an Excel macro implementing the standard FDR algorithm described by Benjamini and Hochberg (1995). Only correlations remaining significant after FDR adjustment were interpreted as robust.

### Statistical analyses

Group differences in EEG power were evaluated for each frequency band and ROI using independent-samples *t*-tests (GD vs. HC). Tests were performed separately for absolute power features and relative power features. Prior to *t*-testing, assumptions of normality and homogeneity of variance were examined. Given that many of the power variables were non-normally distributed, a log transformation or *z*-score standardization was applied as noted above to stabilize variance. Homogeneity of variance was assessed with Levene's test; in cases of violation, Welch's *t*-test (with adjusted degrees of freedom) was used instead of the standard Student's *t*-test (this is indicated in tables by a superscript “a”). All tests were two-tailed with an alpha of 0.05. In addition to *p*-values, effect sizes for group differences were calculated as Cohen's *d* (with pooled SD) to quantify the magnitude of differences. Effect sizes were interpreted as small (~0.2), medium (~0.5), or large (~0.8) as per convention. Complete *t*-test results and effect sizes (Cohen's *d*) for all absolute EEG power features are provided in Supplementary Table S1 Absolute EEG Power. Corresponding results for relative EEG power measures are presented in Supplementary Table S2 Relative EEG Power. Benjamini–Hochberg FDR–adjusted EEG–SOGS correlations are reported in Supplementary Table S3.

To examine the clinical significance of EEG measures, we assessed correlations between EEG power and gambling severity within the GD group. Pearson's correlation coefficients were computed between SOGS scores and the EEG power metrics (both absolute and relative) across GD participants. These analyses were restricted to the GD sample (*n* = 47) to avoid floor effects from HCs with SOGS = 0. A total of 32 correlations were tested for absolute power features and 32 for relative power. Because these multiple comparisons were exploratory, we report nominal *p*-values with significance set at *p* < 0.05 (and note where correlations would survive more stringent thresholds).

All statistical analyses for group comparisons and correlations were carried out in JASP 0.16 and IBM SPSS 27.

### Machine learning classification (LDA)

We employed a supervised machine learning approach—LDA—to investigate the multivariate separability of GD vs. HC based on EEG power features. LDA finds a linear combination of features that best discriminates between classes, producing a linear decision boundary. Two LDA classification models were developed: one using the 32 absolute power features and another using the 32 relative power features. The goal was to determine which feature set (absolute or relative power) yielded better classification performance distinguishing GD patients from HCs.

For each LDA model, the dataset (all 79 subjects) was randomly split into a training set (75% of the data, *n* ≈ 60) and a hold-out test set (25%, *n* = 19). The training set was used to fit the LDA classifier and the independent test set was reserved for evaluating out-of-sample performance. Class priors were set to reflect the training distribution (approximately 0.60 GD, 0.40 HC) and the data were standardized (*z*-scored) feature-wise before model training (as LDA assumes Gaussian features with equal covariance). LDA assumes that input features follow approximately Gaussian distributions with equal class covariances; these assumptions were addressed through *z*-score standardization of all power features before model training, which improves numerical stability and partially satisfies the homoscedasticity requirement. The LDA solution for each model yields a single linear discriminant function (LD1) since there are two classes (GD vs. HC). This function can be expressed by a set of coefficients for each input feature (corresponding to the weight of that standardized feature in the discriminant) and a constant term. These linear discriminant coefficients were extracted to interpret the contribution of each EEG feature to the classification.

**Figure 1:**
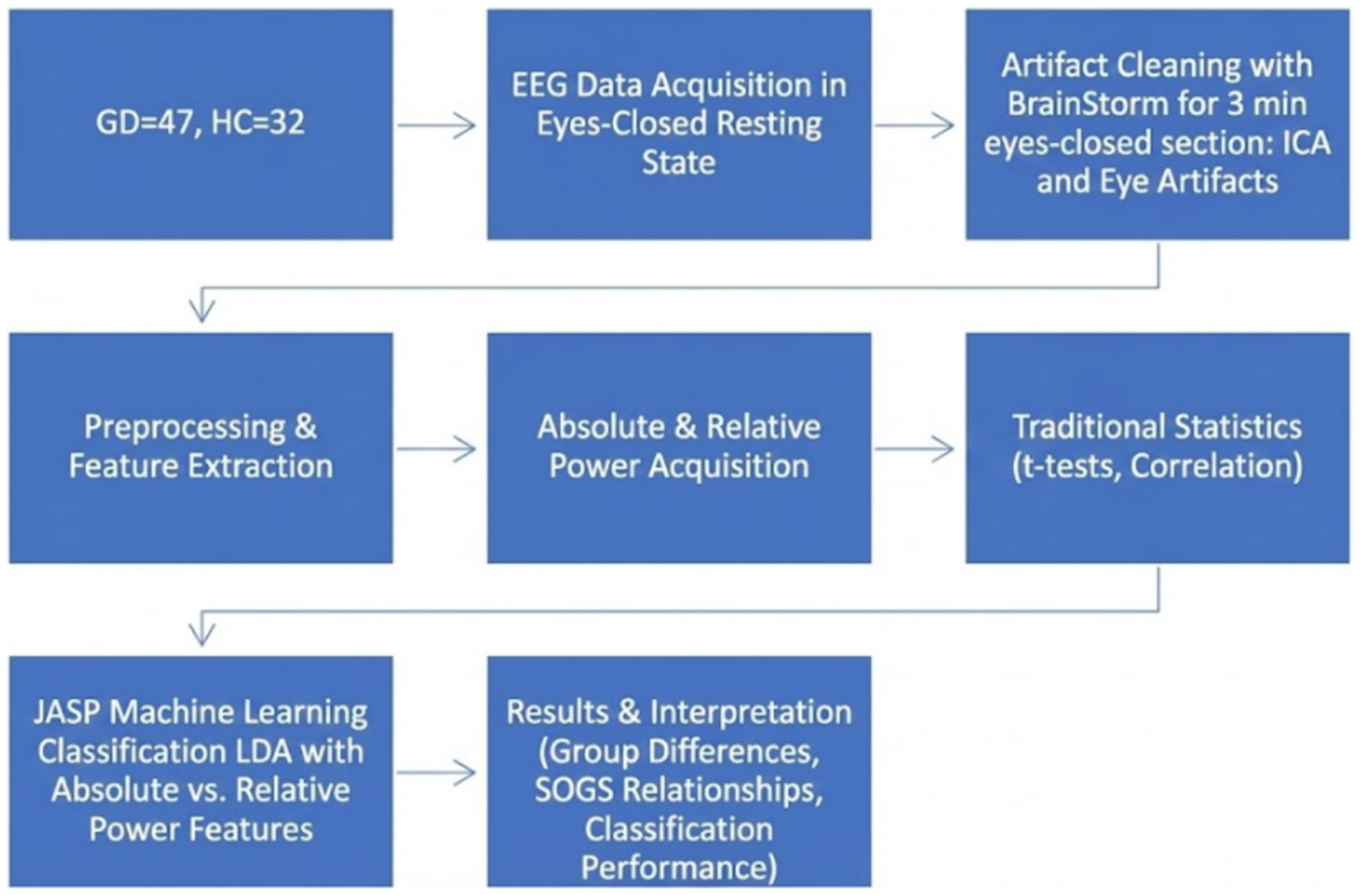
Workflow of EEG data processing, feature extraction, and classification pipeline.

Model performance was evaluated on the test set using several metrics: overall accuracy, precision (positive predictive value) for each class, recall (sensitivity) for each class, F1-score, Matthews correlation coefficient (MCC), and area under the receiver-operating characteristic curve (AUC). Accuracy is the proportion of correct classifications. Precision and recall were computed separately for the GD class (“positive”) and the HC class (“negative”), treating each class as the positive class in turn; F1-score is the harmonic mean of precision and recall. MCC is a balanced measure of binary classification quality (range −1 to +1) that accounts for true and false positives and negatives. AUC was calculated for binary discrimination (considering GD vs. HC) to summarize the sensitivity-specificity trade-off. A confusion matrix was also generated to show the distribution of predicted vs. actual class labels. All performance metrics followed standard definitions (per Scikit-learn/JASP outputs). Model training and evaluation were implemented in JASP's machine learning module, which uses a Fisher's LDA classifier. The trained LDA models were further inspected via plots (e.g., ROC curves, Andrews curves) and the linear discriminant coefficients to facilitate interpretation of the results. An overview of the full methodological pipeline is presented in Figure 1.

This flowchart in Figure 1, illustrates the overall methodology used in the study. Resting-state EEG was recorded from 47 individuals with GD and 32 HC during eyes-closed conditions. Data underwent artifact cleaning in Brainstorm, followed by preprocessing, re-referencing, and extraction of absolute and relative power features across four canonical frequency bands. Traditional statistical analyses (*t*-tests and correlations) were conducted, and LDA was applied to absolute vs. relative power features. Results include group comparisons, SOGS–EEG relationships, and classification performance.

## Results

### Demographics and clinical characteristics

All participants were adult males, and the GD and HC groups were broadly comparable in their demographic characteristics. The mean age of the GD group was 32.5 ± 8.8 years, which was nearly identical to the HC group (32.6 ± 9.9 years), with no significant difference between them (*t*(77) = 0.06, *p* = 0.95). Among GD participants, the average duration of the disorder was 7.03 ± 4.19 years (range: 1–18 years). Regarding psychiatric comorbidity, 13.8% of GD participants (*n* = 4) had at least one co-occurring diagnosis—three individuals (10.3%) had ADHD and one (3.4%) had OCD—whereas no psychiatric diagnoses were present in the HC group, consistent with inclusion criteria. Most GD participants (approximately 79%) had completed at least a high school education, and qualitatively, there were no meaningful group differences in educational attainment. Overall, the groups were well matched on key demographic and clinical variables, minimizing the likelihood of confounding influences on EEG comparisons.

### EEG power: group comparisons (GD vs. HC)

#### Absolute power

**Figure 2:**
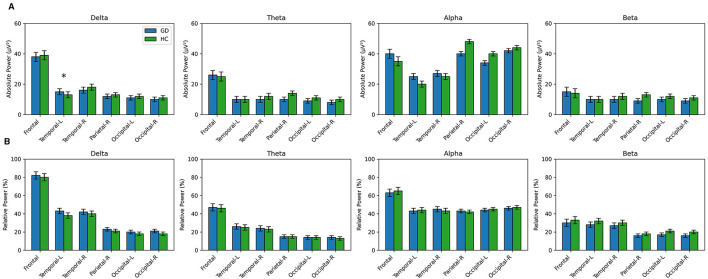
Group mean EEG power across frequency bands and regions in gambling disorder (GD) and healthy controls (HC). **(A)** Absolute EEG power (μV^2^) for delta, theta, alpha, and beta bands across cortical regions. **(B)** Relative EEG power (%) for the same frequency bands and regions. Error bars represent standard error of the mean (SEM).

GD patients and healthy controls showed largely similar resting-state EEG power spectra across most frequency bands and regions, with one notable exception. A significant group difference emerged in the delta band over the left temporal region (Figure 2). GD individuals exhibited higher absolute delta power in left temporal cortex compared to HCs (mean ± SD: GD 15.56 ± 8.04 μV^2^, HC 12.21 ± 7.31 μV^2^), and this difference was statistically significant (*t*(77) = −1.89, *p* = 0.032). The effect size was in the moderate range (Cohen's *d* = 0.43), indicating that GD had moderately greater slow-wave (delta) power in left temporal leads on average. No other absolute power measures differed significantly between groups. In all other regions and frequency bands, the GD group's mean power was comparable to controls (all *p* values > 0.10). For instance, frontal lobe delta and theta power were similar in GD vs. HC (frontal delta: *p* = 0.21; frontal theta: *p* = 0.38), as were parietal and occipital band powers (all *p* > 0.20). The effect sizes for these non-significant differences were generally small (|*d*| < 0.3), suggesting minimal group separation on individual EEG features. Full descriptive statistics and *t*-test results for all absolute power comparisons (GD vs. HC), including Cohen's *d* values, are presented in Supplementary Table S1.

#### Relative power

In contrast to absolute power, relative power metrics did not show any significant group differences. Across all four frequency bands (delta–beta) and all cortical regions, GD and HC groups had statistically equivalent relative power levels (all *p* > 0.11; Supplementary Table S2). For example, the relative delta power in left temporal cortex—which was the lone significant finding in absolute terms—was on average higher in GD (mean ~43.5%) than HCs (~38.0%), but this difference did not reach significance when expressed as a percentage of total power (*p* = 0.115, *d* = 0.37). Similarly, a trend-level difference was observed for relative beta in the left temporal region (GD > HC, *p* = 0.119), but again this did not meet the significance criterion. In general, relative power in each band varied widely between individuals, resulting in considerable overlap between groups and small effect sizes (Cohen's *d* ranged from 0.005 to ~0.36 in absolute magnitude, with no consistent pattern favoring one group). These results indicate that when EEG power is normalized to total spectral power, GD patients do not significantly differ from controls in the distribution of power across delta, theta, alpha, and beta bands. Figure 2 illustrates the group-mean absolute and relative power spectra across brain regions (with error bars), highlighting the absence of robust group differences aside from the noted delta-band finding. Complete results for all relative power comparisons are available in Supplementary Table S2.

Figure 2A displays absolute EEG power (μV^2^) for Delta, Theta, Alpha, and Beta bands across six cortical regions (Frontal, Temporal-L, Temporal-R, Parietal-R, Occipital-L, Occipital-R). Error bars represent SEM. The only significant group difference was observed for left-temporal delta power, where GD showed higher values than HC (*p* = 0.032). No other absolute-power comparisons reached significance. Figure 2B shows relative EEG power (%) for the same bands and regions. No significant group differences were observed for any relative-power measures, consistent with the overall pattern of broadly overlapping GD and HC spectral profiles.

### Correlations of EEG power with gambling severity (SOGS)

Within the GD group, resting EEG power showed several significant associations with gambling severity as measured by SOGS (Table 1). All significant correlations were positive, indicating that higher EEG power in certain bands was associated with higher SOGS scores (greater gambling problem severity). The beta band in particular exhibited the strongest and most consistent correlations with SOGS. In multiple cortical regions, absolute beta power was positively correlated with SOGS scores, with moderate-to-large effect sizes. For example, right parietal beta power had a correlation of *r* = 0.497 (^**^p < 0.001), and bilateral occipital beta power also correlated strongly (*r* ≈ 0.40, *p* = 0.005). Frontal beta power was similarly associated with SOGS (*r* = 0.399, *p* = 0.005 in right frontal) and approached significance in left frontal cortex (*r* = 0.339, *p* = 0.020). These findings suggest that greater high-frequency (beta) activity at rest is linked to greater gambling severity.

**Table 1:** Pearson correlations between absolute EEG power and South Oaks Gambling Screen (SOGS) scores in the gambling disorder group (*n* = 47).

**Region/band**	***r***	***p***	**Region/band**	***r***	***p***
Frontal Δ (L)	0.290	*0.048*	Frontal Δ (R)	0.245	0.097
Frontal θ (L)	0.308	*0.035*	Frontal θ (R)	0.269	0.068
Frontal α (L)	0.248	0.093	Frontal α (R)	0.231	0.119
Frontal β (L)	0.339	*0.020*	Frontal β (R)	0.399	**0.005**
Temporal Δ (L)	0.239	0.106	Temporal Δ (R)	0.273	0.063
Temporal θ (L)	0.213	0.150	Temporal θ (R)	0.261	0.076
Temporal α (L)	0.251	0.089	Temporal α (R)	0.325	*0.026*
Temporal β (L)	0.381	**0.008**	Temporal β (R)	0.446	**0.002**
Parietal Δ (L)	0.280	0.057	Parietal Δ (R)	0.383	**0.008**
Parietal θ (L)	0.243	0.100	Parietal θ (R)	0.354	*0.015*
Parietal α (L)	0.230	0.121	Parietal α (R)	0.288	*0.050*
Parietal β (L)	0.376	**0.009**	Parietal β (R)	0.497	*** < 0.001***
Occipital Δ (L)	0.288	*0.049*	Occipital Δ (R)	0.167	0.261
Occipital θ (L)	0.297	*0.042*	Occipital θ (R)	0.289	*0.049*
Occipital α (L)	0.327	*0.025*	Occipital α (R)	0.259	0.078
Occipital β (L)	0.403	**0.005**	Occipital β (R)	0.404	**0.005**

Values represent Pearson correlation coefficients (r) and associated *p*-values between EEG absolute power (μV^2^) in each band/region and SOGS scores within the GD group (n = 47). No significant correlations were observed for relative power measures.

Bold values indicate significance: *p* < 0.05; ^**^p < 0.01; ^***^p < 0.001. Italic values indicate trend-level associations (0.05 < *p* < 0.10).

The alpha band also showed notable positive correlations with SOGS in some regions. For instance, absolute alpha power in the right temporal region was moderately correlated with SOGS (*r* = 0.325, *p* = 0.026). Likewise, occipital alpha power (particularly left occipital) was significantly associated with SOGS (*r* = 0.327, *p* = 0.025). Parietal alpha showed a trend-level correlation (*r* = 0.288, *p* = 0.050 for right parietal). Thus, individuals with higher resting alpha power in posterior regions tended to report higher gambling severity.

**Figure 3:**
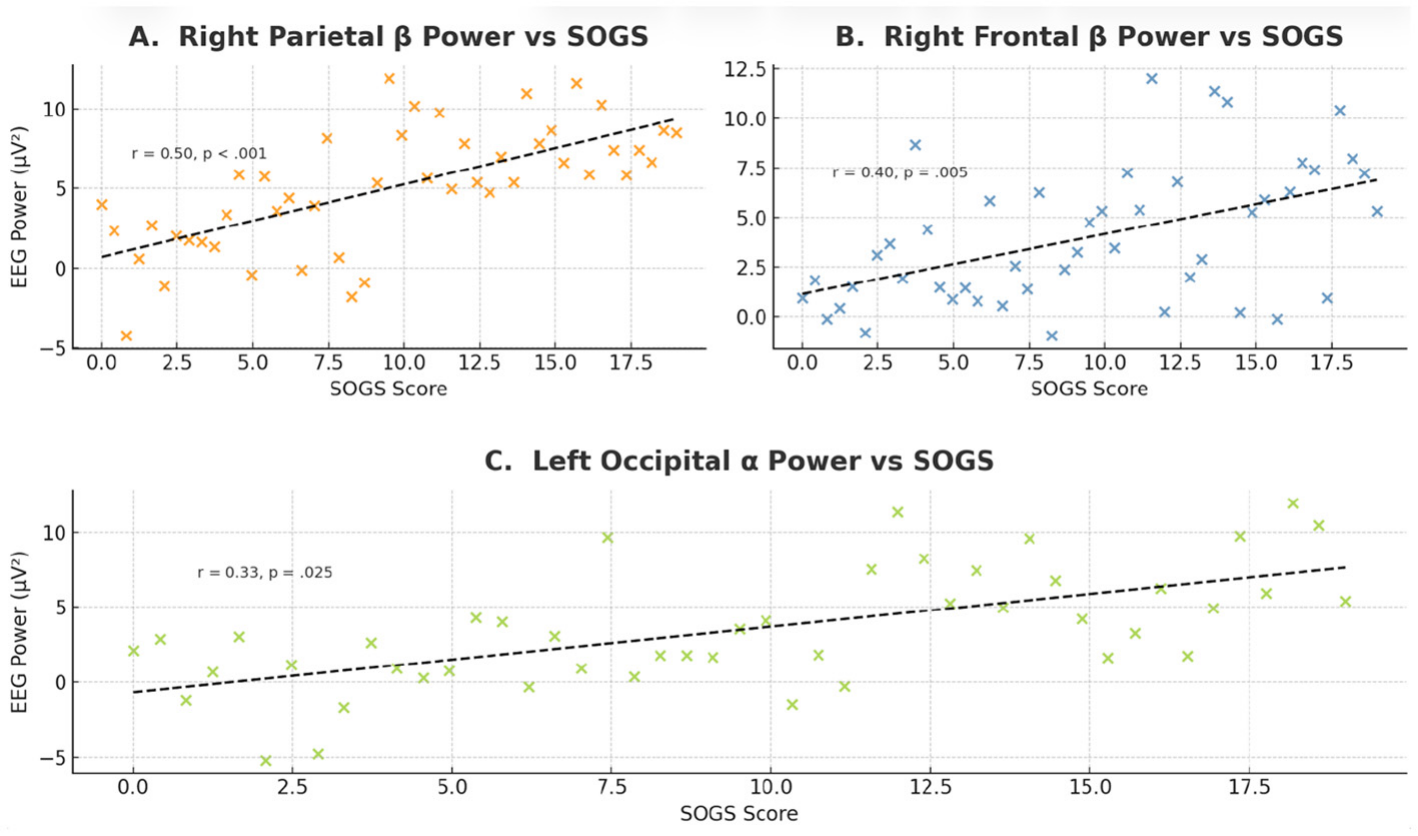
Associations between EEG power and gambling severity (SOGS scores). **(A)** Right Parietal β Power vs. SOGS, **(B)** Right Frontal β Power vs. SOGS and **(C)** Left Occipital β Power vs. SOGS.

For lower-frequency bands (delta and theta), correlations with SOGS were generally weaker, but a few reached significance in frontal regions. Notably, left frontal delta and theta power were moderately correlated with SOGS (delta: *r* = 0.290, *p* = 0.048; theta: *r* = 0.308, *p* = 0.035). This implies that GD patients with more pronounced slow-wave (delta/theta) activity in frontal cortex tended to have higher SOGS scores. Additionally, right parietal delta and theta showed positive trends (*r* ≈ 0.35 for theta, *p* = 0.015; *r* = 0.383 for delta, *p* = 0.008), and left occipital delta approached significance (*r* = 0.274, *p* = 0.062). Although not all of these survived correction for multiple comparisons, the overall pattern indicates that higher power across several bands—most consistently beta—correlates with more severe gambling symptoms. Importantly, no significant correlations were found between SOGS and any relative power measures (all *p* > 0.1), suggesting that the relationships with clinical severity are specific to absolute EEG power rather than the proportional distribution of power. All EEG–SOGS correlation results, including the FDR-adjusted values, are provided in Supplementary Table S3, and key relationships (such as beta power vs. SOGS) are shown with scatter plots in Figure 3.

To control for Type I error inflation due to multiple testing, the Benjamini–Hochberg FDR correction was applied to all EEG–SOGS correlations. After correction, the majority of strong beta-band correlations remained significant, confirming their robustness. Specifically, right parietal beta (*p*FDR = 0.008), right frontal beta (*p*FDR = 0.019), and bilateral occipital beta (*p*FDR = 0.027) associations persisted after FDR adjustment, whereas weaker delta, theta, and alpha correlations no longer met the corrected threshold. These results indicate that the most reliable EEG–severity relationships were in the beta band, highlighting its potential as an electrophysiological marker of gambling problem severity. No significant FDR-corrected correlations were observed for relative power measures (all *p*FDR > 0.10), suggesting that these effects are specific to absolute power rather than proportional spectral composition. Full unadjusted and FDR-adjusted correlation values are provided in Supplementary Table S3. All EEG–SOGS correlations with Benjamini–Hochberg FDR–adjusted *p*-values are provided in Supplementary Table S3.

In Figure 3, scatterplots display the significant positive correlations between absolute EEG band power and South Oaks Gambling Screen (SOGS) scores in the GD group (*n* = 47). Figure 3A: right parietal β power (*r* = 0.50, *p* < 0.001), Figure 3B: right frontal β, power (*r* = 0.40, *p* = 0.005), Figure 3C: left occipital α power (*r* = 0.33, *p* = 0.025). Regression lines (black dashed) represent least-squares fits; each point denotes a participant. Higher EEG power correlated with greater gambling severity, especially within β- and α-band posterior regions.

### LDA classification of GD vs. HC

Using multivariate EEG features, the LDA classifiers were able to distinguish GD patients from healthy controls with moderate success. Classification performance differed substantially depending on whether absolute power or relative power features were used.

#### LDA with absolute power features

**Figure 4:**
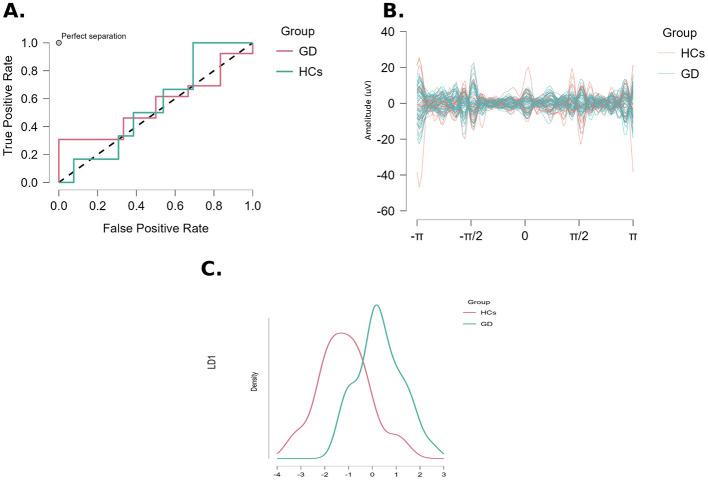
Linear discriminant analysis (LDA) classification performance using absolute EEG power features. **(A)** Receiver operating characteristic (ROC) curve for the LDA model distinguishing GD from HC, showing the area under the curve (AUC). **(B)** Andrews curves illustrating multivariate feature separation between GD and HC groups. **(C)** Density distribution of linear discriminant (LD) scores for GD and HC participants.

The LDA model based on the 32 absolute power features achieved an overall accuracy of 73.7% in classifying the hold-out test set (14 out of 19 test subjects correctly classified). This represents a considerable improvement over chance (50%) and indicates that absolute EEG power contains discriminatory information for GD vs. HC. The confusion matrix for this model is illustrated in Figure 4. In the test set, 10 of 11 GD participants were correctly predicted as “GD” (true positive rate/sensitivity = 90.9%), while four of eight HCs were correctly identified as “HC” (true negative rate/specificity = 50.0%). In other words, the classifier tended to be biased toward the GD class, yielding a few false positives (four HCs misclassified as GD) but very few false negatives (only one GD misclassified as HC). The precision for the GD class was 71.4%, meaning that 10/14 subjects predicted as GD were true positives, whereas the precision for the HC class was 80.0% (4/5 predicted HCs were actual HCs). The imbalance in sensitivity vs. specificity reflects that the model was more optimized to catch GD cases at the expense of falsely labeling some HCs as gamblers.

Other performance metrics indicated good discriminative ability of the absolute-power LDA. The F1-scores were 0.80 for the GD class and 0.62 for the HC class, with a macro-averaged F1 of 0.72. The Matthews correlation coefficient (MCC) for the model was 0.459, which is considered a moderate level of overall classification quality (with 0 indicating no better than chance). The area under the ROC curve (AUC) was 0.74, confirming that the model achieved substantially better-than-chance discrimination between GD and HC (AUC of 0.5 represents chance level). In sum, using absolute EEG power features, the LDA classifier reached nearly 74% accuracy with robust sensitivity for detecting GD. This suggests that multi-feature EEG profiles (even in resting state) carry a signal that differentiates pathological gamblers from non-gamblers, although specificity was more moderate. Notably, the only EEG feature that was individually significant (left temporal delta) was not solely responsible for this accuracy; rather, the combination of numerous features contributed to the classification.

Analysis of the LDA model's coefficients provides insight into which EEG features were most influential in separating the groups. The linear discriminant coefficients are essentially the weights of each *z*-scored feature in the discriminant function LD1. We observed that theta-band powers had some of the largest coefficient magnitudes, indicating a strong role in the discriminative direction. For example, right parietal theta power had one of the highest positive coefficients (approximately +8.84), and left parietal theta had a large negative coefficient (~-5.73). Likewise, occipital theta coefficients were prominent (e.g., +5.35 for left occipital theta, −5.05 for right occipital theta). These large-magnitude weights suggest that differences in theta activity across parietal-occipital regions contribute substantially to distinguishing GD from HC in the LDA space. In contrast, many beta-band features had relatively small coefficients (generally < |2.0|), consistent with the univariate finding that group means in beta power were not significantly different. Interestingly, frontal theta (coefficient ~+5.13 for right frontal theta) and frontal delta (−1.48 for right frontal delta, +1.17 for left frontal delta) also had notable weights, aligning with the idea that slow-wave activity, especially in frontal and parietal areas, differentiated the groups to some extent. The left temporal delta feature (the only univariate significant marker) had a moderate positive coefficient (~+1.39), indicating that higher left temporal delta tended to push the discriminant score toward the GD class—in agreement with the group difference. Overall, the pattern of coefficients suggests the LDA model identified a multivariate signature of GD characterized by a complex interplay of increased theta (and to a lesser extent delta) in certain regions and subtle shifts in alpha/beta, rather than relying on any single power measure. These coefficients provide a basis for neurophysiological interpretation of the classification: they point toward widespread cortical oscillatory alterations (notably in the theta band) associated with GD.

#### LDA with relative power features

When relative power features were used as predictors, classification performance dropped markedly. The LDA model using the 32 relative power features achieved a test accuracy of only 57.9% (11/19 correct), which is only slightly above chance level (50%). This indicates that relative power measures were far less effective in discriminating GD from HCs. The confusion matrix for the relative-power LDA revealed an imbalance in classifications: the model correctly identified 10 out of 13 GD cases in the test set (sensitivity = 76.9%) but correctly identified only one out of six HCs (specificity = 16.7%). In fact, the classifier using relative features tended to misclassify the majority of healthy controls as gamblers. This bias led to a high false-positive rate (83% of HCs were classified as GD) and a poor precision for the HC class (only 25% of those predicted as HC were actual HC). The precision for the GD class was 66.7%, reflecting the model's tendency to over-predict the GD category. The F1-score for GD was 0.71, but for HCs it was an extremely low 0.20, underscoring that the model performed very unevenly between classes. The overall MCC was −0.073, essentially ~0, indicating no meaningful skill in classification when considering both sensitivity and specificity. The AUC was 0.55, which is only slightly above chance and much lower than the AUC for the absolute-power model. In summary, the LDA based on relative power failed to reliably distinguish gamblers from controls; it defaulted to classifying most subjects as GD, capturing true positives at the expense of an unacceptable false-positive rate. This outcome aligns with the univariate results where relative power showed no significant group differences—implying that the proportional makeup of the EEG spectrum does not provide a stable signature of GD.

## Discussion

In this study, we investigated resting-state EEG biomarkers of GD using both traditional spectral analysis and a machine learning classification approach. The overall pattern of findings indicates that baseline EEG power differences between GD patients and healthy controls are limited in scope, with most spectral measures showing substantial overlap between groups. The only statistically significant group difference emerged in the left temporal delta band, suggesting that any resting-state neural alterations associated with GD may be subtle and regionally circumscribed rather than widespread. At the same time, several EEG features—particularly absolute beta power across posterior regions—showed meaningful associations with gambling severity within the GD cohort, indicating that resting EEG may still carry clinically relevant information even when case–control differences are weak. Consistent with this interpretation, the multivariate LDA classifier achieved modest above-chance accuracy, implying that distributed patterns of EEG activity, rather than single features, may contribute to distinguishing individuals with GD from healthy controls. Taken together, these results suggest that GD may not produce uniform or pronounced deviations in resting-state EEG power but may instead involve small, spatially specific oscillatory differences and within-group variability that relate to disorder severity.

The lack of broad between-group EEG differences is an interesting outcome that warrants careful reflection. We had hypothesized that GD subjects would show elevated delta/theta and reduced beta power relative to controls, in line with some prior reports and the well-described neurocognitive profile of GD involving impulsivity and possible cortical underactivation. Instead, for most brain regions and frequency bands, GD patients' resting EEG power was statistically indistinguishable from that of healthy individuals. This largely negative pattern is partly consistent with earlier work; for instance, Lee et al. (2017) also reported no significant resting-state spectral or hemispheric abnormalities in pathological gamblers, suggesting that baseline cortical activity in GD can appear within normative ranges. One possibility is that neurophysiological alterations in GD are subtle, highly variable across individuals, or spatially restricted, making them difficult to detect with standard univariate metrics in a moderately sized sample. It may also be that the neural disruptions characteristic of GD emerge more clearly during gambling-related or emotionally arousing contexts—such as reward processing, decision-making, or loss anticipation—rather than under eyes-closed resting conditions. Moreover, our GD sample displayed heterogeneity in clinical features despite meeting diagnostic criteria, and this variability may have introduced noise that obscured group-level effects. Although none of the participants were taking psychotropic medication, several had mild comorbidities (e.g., ADHD, OCD), and such conditions can modulate frontal slow-wave or theta–beta dynamics in different directions; when averaged, these influences may counterbalance one another and reduce detectable group differences. These considerations underscore the need for future studies with larger samples, more homogeneous clinical subgroups, and task-based or stress-induced EEG paradigms, which may be more sensitive to the neurophysiological signatures of gambling disorder.

Nonetheless, the one significant group difference we observed—increased delta power over the left temporal region in GD patients—is noteworthy and aligns with prior reports of localized EEG abnormalities in gambling populations. Temporal slow-wave activity has frequently been described as a characteristic irregularity in pathological gamblers (Qi et al., 2022). Consistent with this, Regard et al. (2003) found that in gamblers with abnormal EEGs, the majority exhibited focal temporal slowing. Our finding of heightened delta (1–4 Hz) power in left temporal leads echoes this pattern and may point to a region-specific neural alteration in GD. The temporal lobe encompasses structures involved in memory, emotion, and aspects of reward learning (Jacobson et al., 2025), domains often implicated in addictive behaviors. Excess slow-wave activity in this area may therefore reflect underlying functional impairments or altered neuromodulatory processes—such as serotonergic or dopaminergic modulation—associated with chronic gambling behavior (Potenza, 2013). Alternatively, increased delta activity could indicate a state of cortical hypoactivation or maturational lag, as diffuse delta oscillations are often interpreted as markers of reduced neuronal firing or cortical idling (Balconi et al., 2015). While our data cannot determine the precise origins of this effect, its localization supports the possibility that GD is associated with subtle electrophysiological footprints in brain regions linked to addiction circuitry (Clark et al., 2019). Future work using high-density EEG, source localization, or multimodal imaging may help clarify whether this temporal delta elevation reflects deeper limbic involvement—possibly related to craving or affective dysregulation—or instead reflects localized cortical network alterations.

The correlation analyses offer additional insight into the neurophysiology of GD by linking specific EEG features to clinical severity. Participants with more severe gambling problems (higher SOGS scores) tended to show higher absolute power in several frequency bands, with the most consistent effects emerging in the beta range across frontal, temporal, parietal, and occipital sites. This pattern aligns with reports by Kim et al. (2018, 2019), who likewise observed positive associations between beta activity and gambling severity. Elevated beta power is often interpreted as an index of heightened cortical arousal or excitability (Clarke et al., 2019), and our findings are compatible with the idea that more severe GD is characterized by a state of increased neural activation at rest (Chmiel and Malinowska, 2025). Clinically, such a pattern may reflect greater craving, stress responsivity, or impulsive drive in individuals with more entrenched gambling behavior, consistent with prior suggestions that beta elevations may capture a state of hyperexcitability and craving in severe gamblers.

Another possible interpretation is that individuals with more severe GD exhibit neurophysiological features shared with other impulsivity-related conditions (Kaasinen et al., 2023). For example, ADHD and related high-impulsivity profiles often show reduced beta and elevated theta activity, yielding a high theta/beta ratio (Loo et al., 2013). Although we did not observe a theta/beta ratio effect *per se*, we did find that frontal delta and theta power showed modest positive correlations with gambling severity. Such slow-wave activity in frontal regions is frequently associated with reduced executive control or cognitive fatigue (Diep et al., 2020), and its presence among higher-severity gamblers may reflect weakening prefrontal regulatory mechanisms as gambling problems intensify (de Ruiter et al., 2012). Thus, a plausible interpretation is that increasing GD severity may be accompanied by a dual pattern: heightened beta activity reflecting arousal or craving (Fox et al., 2012) alongside increased frontal slow-wave activity indicative of diminished executive oversight (Lucchiari and Pravettoni, 2010). Together, these oscillatory shifts could contribute to a neurophysiological profile characterized by simultaneous overactivation and underregulation, creating conditions favorable for loss of behavioral control in severe GD. Importantly, the positive associations between beta power and gambling severity remained significant after Benjamini–Hochberg FDR correction, underscoring the robustness of this relationship and reducing the likelihood that these findings reflect chance.

It is worth noting that not all of our EEG–severity correlations align neatly with prior research. For example, we found that higher alpha power in right temporal and occipital regions was associated with greater gambling severity. Alpha activity (8–13 Hz) is typically considered the dominant rhythm of relaxed wakefulness (Cantero et al., 2002) and is often interpreted as an index of cortical idling or inhibition of task-irrelevant processing (Uusberg et al., 2013). Based on this framework, one might expect individuals with more severe gambling problems to exhibit reduced alpha—reflecting heightened stress, craving, or arousal (de Castro et al., 2007). Instead, our findings suggest the opposite: more severe gamblers showed stronger posterior alpha. Interestingly, Lee et al. (2019) reported a similar paradoxical pattern, observing elevated relative alpha power in GD patients in a precontemplation stage, which they interpreted as reflecting a withdrawn or internally focused state. From this perspective, increased alpha may index a form of disengagement or experiential “retreat,” in which individuals remain in a familiar cognitive–emotional stance despite escalating gambling problems. In our sample, higher temporal and occipital alpha among more severe GD cases could similarly reflect a tendency toward internally oriented attention or a relaxed, possibly dissociative mental state (Griffiths et al., 2006; Rogier et al., 2021) as gambling severity increases. Neurochemical factors may also play a role: alpha oscillations are modulated by GABAergic and cholinergic systems (Liljenstrom and Hasselmo, 1995; Pafundo et al., 2013), both of which can be altered in chronic addictive behaviors. These converging interpretations suggest that alpha abnormalities may not reflect simple reductions in arousal but could represent a subtype or state-dependent neural response in GD—for example, individuals who are less anxious, more avoidant, or less motivated to change. Taken together, and consistent with previous literature (Son et al., 2015; Kornev et al., 2025; Newson and Thiagarajan, 2019), our results highlight that resting-state EEG measures can be meaningfully related to clinical characteristics of GD even when case–control differences are subtle. They may capture a continuum of dysfunction, where the magnitude of neural deviation scales with gambling severity rather than simply distinguishing GD from non-GD individuals. In this sense, EEG could have potential utility as an adjunctive indicator of problem gambling severity (Megías et al., 2018), complementing self-report measures and offering an objective index of symptom burden.

A major component of our study was the application of a supervised machine learning classifier (LDA) to the EEG data (Bhardwaj et al., 2015). The LDA model based on absolute power features achieved an overall accuracy of approximately 74% in distinguishing GD patients from healthy individuals (Seo et al., 2020), significantly above chance. Although this represents only moderate performance, it is notable given the limited number of univariate group differences described earlier. This suggests that a linear combination of EEG features can encode diagnostic information about GD (Yeh et al., 2025) that is not evident when examining individual variables in isolation. Indeed, the success of the classifier highlights that GD may differ from HC not through a single dominant spectral abnormality, but rather through a distributed pattern of oscillatory characteristics (Li et al., 2019). Examination of discriminant coefficients revealed that the theta band contributed most strongly to group separation, particularly within parietal, occipital, and frontal regions, implying that alterations in theta dynamics across multiple cortical areas may be especially relevant for identifying GD (Gomis-Vicent, 2021). More broadly, the model appeared to rely on a subtle interplay of elevated theta—and to a lesser extent delta—in certain regions (Lee et al., 2024), accompanied by weaker shifts in alpha and beta activity. This pattern is consistent with our overall hypothesis that GD involves distributed, small-scale oscillatory deviations that may serve as a multivariate “fingerprint” of the disorder. Clinically, the high sensitivity of the model (~91%) is encouraging, as it indicates that most GD individuals were correctly identified (Khajehpour et al., 2019). However, the modest specificity (~50%) indicates a tendency to misclassify healthy individuals as GD, suggesting that the classifier may over-detect pathological patterns when group differences are subtle. Such a bias limits the utility of this approach as a stand-alone screening tool and underscores the need for more balanced models. Nonetheless, as a proof of concept, our findings demonstrate that resting-state EEG does contain discernible multivariate information relevant to GD diagnosis. This is consistent with broader trends in psychiatric research, where machine learning methods have increasingly been applied to EEG and other neurobiological data to identify patterns that escape traditional analyses (Hosseini et al., 2020; Katinni et al., 2024). Looking ahead, predictive performance might be improved with larger samples, more sophisticated algorithms (e.g., nonlinear classifiers or deep learning; Le, 2015), or multimodal feature integration. Such refinements may ultimately help define more reliable EEG-based markers of GD and enhance the generalizability of classification models across diverse populations.

It is also informative to compare the performance of absolute vs. relative power features in our classification task. The LDA model trained on relative power—each band expressed as a percentage of total spectral power—performed only slightly above chance (~58% accuracy), in clear contrast to the absolute power model (~74% accuracy). This discrepancy suggests that the discriminative signal in resting-state EEG is carried primarily by absolute amplitude differences rather than by the normalized spectral profile. Put differently, pathological gamblers and healthy controls did not differ meaningfully in the proportional distribution of delta, theta, alpha, or beta power across regions, a pattern consistent with our univariate findings showing no significant group differences in relative power. Instead, group separation appeared to emerge from small absolute shifts in power that varied across bands and regions. When total power is normalized out, these subtle magnitude differences are likely dampened or eliminated, reducing classification performance. From a physiological standpoint, one interpretation is that GD may involve slight increases or decreases in absolute power across specific frequency ranges—such as modest elevations in theta—without altering the overall balance between frequency bands. Such global or regionally specific amplitude shifts would influence absolute values but would have minimal impact on relative percentages. This possibility highlights the importance of examining both absolute and relative measures: although relative power is widely used in psychiatric EEG research, it may obscure small but meaningful between-group differences in spectral magnitude. Accordingly, our findings suggest that researchers should be cautious about relying solely on normalized spectral measures, as critical information relevant to case–control discrimination may reside in total power levels even when spectral ratios appear unchanged.

### Clinical and research implications

The present findings, while preliminary, contribute to the growing understanding of GD's neurobiology and have several potential implications. Clinically, the relative normality of resting-state EEG in GD—with the exception of specific slow-wave increases—suggests that baseline cortical function may remain largely intact in many individuals with the disorder. This could help explain why GD often remains undetected unless behavioral or functional impairments become overt (Potenza et al., 2019), as spontaneous resting activity may not deviate strongly from the healthy range. At the same time, the observed correlations between EEG power and gambling severity indicate that resting EEG may still capture clinically meaningful variation. Individuals with GD who exhibit especially high beta power alongside elevated delta/theta activity might represent cases with more entrenched symptomatology or heightened craving and stress, raising the possibility that EEG-based indices could complement self-report measures for severity stratification or treatment monitoring. These findings also raise questions regarding potential neuromodulatory or neurofeedback approaches. If future research confirms that increased slow-wave activity in temporal or frontal regions is a reliable feature of GD, training protocols aimed at reducing slow oscillations—or enhancing faster rhythms—may have therapeutic relevance. Such approaches parallel neurofeedback strategies used in other conditions involving impulsivity and dysregulation, such as ADHD and certain substance use disorders. Although the present results do not establish causality, they provide an initial rationale for exploring whether EEG-guided interventions could help modify neural patterns associated with severe gambling behavior.

From a research perspective, the moderate but above-chance performance of the LDA classifier demonstrates that resting EEG contains measurable information relevant to distinguishing GD from healthy individuals. While ~74% accuracy does not support clinical utility at this stage, it suggests that multivariate signatures—and not only isolated spectral features—carry diagnostic relevance. Future studies with larger and more diverse samples could apply more advanced machine-learning methods (e.g., support vector machines, neural networks) with rigorous cross-validation to better characterize these signatures. Incorporating additional EEG features such as coherence, functional connectivity, or event-related responses may further improve discrimination, given prior evidence of altered connectivity patterns in gamblers. Comparative studies across behavioral addictions (e.g., gambling vs. internet gaming disorder) could also clarify whether certain oscillatory markers, such as theta abnormalities, represent shared or disorder-specific neurophysiological features. Finally, longitudinal research is needed to determine whether the EEG characteristics observed here—such as elevated beta or slow-wave activity—are stable correlates of symptom severity or predictive markers of future outcomes, including relapse or response to treatment. If these measures prove reliable across time, resting EEG could eventually play a role in identifying high-risk individuals, informing personalized interventions, or tracking clinical progress. In this sense, the present findings serve as an early foundation for a more nuanced and clinically integrated understanding of EEG biomarkers in gambling disorder.

### Limitations

We acknowledge several limitations of our study. The sample size (47 GD patients and 32 controls) was relatively modest for detecting small EEG effects and for training a classifier—this may have limited our power to find group differences and constrained the machine learning model's performance. Because the entire sample consisted of adult males, the results cannot be generalized to female gamblers or to mixed-gender populations. Future research including balanced male and female samples is needed to determine whether the EEG patterns observed here also characterize women with Gambling Disorder. We did not control for potential confounders like caffeine use, time of day, or recent gambling activity, which might influence resting EEG (e.g., acute stress from a gambling episode could elevate beta). The GD group had a few comorbid diagnoses (e.g., ADHD in ~10% of patients); although this reflects real-world clinical heterogeneity, it muddles interpretation since ADHD is known to affect EEG (typically increasing theta). Our analysis approach, particularly doing multiple *t*-tests and correlations, also comes with an inherent risk of Type I error. We chose an exploratory threshold of *p* < 0.05 without formal correction due to the novel, hypothesis-generating nature of this work—thus some findings (such as the single significant group difference) should be considered tentative until replicated. Similarly, the LDA classifier was evaluated on a hold-out set but not on an independent replication sample; its performance might be optimistic and could vary with different train-test splits. We mitigated overfitting by using a simple model and few features (relative to data points), but a degree of overfitting remains possible given the high dimensionality of the EEG feature space. Future studies should employ cross-validation or external validation samples to verify classification results.

## Conclusion

This research offers a deeper look into the resting-state EEG characteristics of GD and illustrates how combining traditional analysis with machine learning can yield insights into a complex psychiatric condition. We found that GD, as a group, is not marked by dramatic abnormalities in resting EEG power—a result that tempers expectations of using EEG for easy case identification. However, more subtle patterns emerged: GD patients showed a localized increase in slow-wave (delta) activity and multiband EEG changes correlating with clinical severity, suggesting that the brain's electrical activity does track the extent of gambling pathology. Moreover, by harnessing a multivariate classifier, we uncovered an EEG-derived signature that differentiates gamblers from non-gamblers with moderate accuracy, highlighting the promise of data-driven approaches in psychiatric diagnosis. These findings reinforce the view of GD as a disorder of brain function that might not be obvious on gross examination but can be detected through careful quantitative measures. Going forward, replication and expansion of this work are needed—including larger and more diverse populations, exploration of EEG connectivity metrics, and integration with other modalities (such as MRI or genetic data)—to fully establish the neurophysiological underpinnings of GD. Such efforts will ultimately enhance our ability to identify, monitor, and treat GD, moving toward a more objective, brain-based understanding of addiction.

## Data Availability

The original contributions presented in the study are included in the article/Supplementary material, further inquiries can be directed to the corresponding author.
