# Supplementary material for: Resting-state EEG power and machine-learning classification in adult males with gambling disorder
Source: Front Hum Neurosci. 2026 Jan 13;19:1725528. doi: 10.3389/fnhum.2025.1725528 (PMC12835224; doi:10.3389/fnhum.2025.1725528)
Supplement: Supplementary file 1 [file Table_1.docx]

S1- Absolute EEG Power (µV²) Comparisons between Gambling Disorder (GD) and Healthy Controls (HC)

| **Region / Band** | **Group** | **Mean ± SD** | **t (df = 77)** | **p** | **Cohen’s *d*** |
| --- | --- | --- | --- | --- | --- |
| **Frontal Δ** | GD | 38.65 ± 20.74 | –0.01 | 0.496 | –0.00 |
|  | HC | 38.60 ± 21.03 |  |  |  |
| **Frontal θ** | GD | 26.27 ± 18.67 | –0.31 | 0.380 | –0.07 |
|  | HC | 24.67 ± 27.63 |  |  |  |
| **Frontal α** | GD | 40.17 ± 41.75 | –0.59 | 0.279 | –0.14 |
|  | HC | 35.14 ± 29.25 |  |  |  |
| **Frontal β** | GD | 14.84 ± 8.32 | –0.53 | 0.300 | –0.12 |
|  | HC | 13.93 ± 6.09 |  |  |  |
| **Temporal Δ (L)** | GD | 15.56 ± 8.04 | –1.89 | **0.032** | 0.43 |
|  | HC | 12.21 ± 7.31 |  |  |  |
| **Temporal θ (L)** | GD | 10.13 ± 6.80 | –0.19 | 0.424 | –0.04 |
|  | HC | 9.72 ± 12.13 |  |  |  |
| **Temporal α (L)** | GD | 24.36 ± 27.79 | –0.87 | 0.194 | –0.20 |
|  | HC | 19.59 ± 16.73 |  |  |  |
| **Temporal β (L)** | GD | 10.01 ± 6.03 | 0.03 | 0.511 | 0.01 |
|  | HC | 10.04 ± 5.54 |  |  |  |
| **Temporal Δ (R)** | GD | 14.95 ± 12.82 | 0.58 | 0.719 | 0.13 |
|  | HC | 16.91 ± 17.07 |  |  |  |
| **Temporal θ (R)** | GD | 9.95 ± 7.91 | 0.63 | 0.734 | 0.14 |
|  | HC | 12.03 ± 20.65 |  |  |  |
| **Temporal α (R)** | GD | 26.94 ± 25.13 | –0.24 | 0.406 | –0.06 |
|  | HC | 25.58 ± 24.26 |  |  |  |
| **Temporal β (R)** | GD | 10.11 ± 7.08 | 1.37 | 0.912 | 0.31 |
|  | HC | 12.57 ± 8.85 |  |  |  |
| **Parietal Δ (R)** | GD | 11.90 ± 6.73 | 0.70 | 0.756 | 0.16 |
|  | HC | 12.94 ± 6.20 |  |  |  |
| **Parietal θ (R)** | GD | 9.81 ± 6.98 | 1.02 | 0.845 | 0.23 |
|  | HC | 14.65 ± 31.36 |  |  |  |
| **Parietal α (R)** | GD | 39.53 ± 42.39 | 0.79 | 0.785 | 0.18 |
|  | HC | 48.94 ± 63.27 |  |  |  |
| **Parietal β (R)** | GD | 9.59 ± 6.46 | 1.54 | 0.937 | 0.35 |
|  | HC | 13.20 ± 13.98 |  |  |  |
| **Occipital Δ (L)** | GD | 10.38 ± 6.24 | 0.80 | 0.787 | 0.18 |
|  | HC | 11.63 ± 7.60 |  |  |  |
| **Occipital θ (L)** | GD | 8.52 ± 6.09 | 0.80 | 0.786 | 0.18 |
|  | HC | 10.49 ± 15.36 |  |  |  |
| **Occipital α (L)** | GD | 34.33 ± 43.15 | 0.55 | 0.706 | 0.13 |
|  | HC | 39.51 ± 38.76 |  |  |  |
| **Occipital β (L)** | GD | 10.02 ± 8.81 | 1.04 | 0.848 | 0.24 |
|  | HC | 12.19 ± 9.64 |  |  |  |
| **Occipital Δ (R)** | GD | 10.19 ± 5.66 | 0.58 | 0.718 | 0.13 |
|  | HC | 11.10 ± 8.34 |  |  |  |
| **Occipital θ (R)** | GD | 8.21 ± 5.77 | 0.77 | 0.778 | 0.18 |
|  | HC | 10.33 ± 17.61 |  |  |  |
| **Occipital α (R)** | GD | 41.61 ± 50.16 | 0.16 | 0.565 | 0.04 |
|  | HC | 43.43 ± 47.04 |  |  |  |
| **Occipital β (R)** | GD | 8.80 ± 5.64 | 1.86 | 0.967 | 0.43 |
|  | HC | 11.81 ± 8.74 |  |  |  |

**Note.** Values represent mean ± SD absolute EEG power (µV²) in each frequency band and ROI for GD and HC groups. Negative *t* values reflect higher mean power in GD.
Bold *p* values (< 0.05) denote significant group differences (only left-temporal delta).
