# Supplementary material for: Resting-state EEG power and machine-learning classification in adult males with gambling disorder
Source: Front Hum Neurosci. 2026 Jan 13;19:1725528. doi: 10.3389/fnhum.2025.1725528 (PMC12835224; doi:10.3389/fnhum.2025.1725528)
Supplement: Supplementary file 2 [file Table_2.docx]

S2-Relative EEG Power (%) Comparisons between Gambling Disorder (GD) and Healthy Controls (HC)

| **Region / Band** | **Group** | **Mean ± SD** | **t (df = 77)** | **p** | **Cohen’s *d*** |
| --- | --- | --- | --- | --- | --- |
| **Frontal Δ** | GD | 80.86 ± 26.92 | –0.18 | 0.861 | –0.04 |
|  | HC | 79.32 ± 21.01 |  |  |  |
| **Frontal θ** | GD | 47.39 ± 12.52 | –0.29 | 0.770 | –0.07 |
|  | HC | 46.52 ± 13.79 |  |  |  |
| **Frontal α** | GD | 63.09 ± 33.33 | –0.11 | 0.913 | –0.03 |
|  | HC | 63.46 ± 29.95 |  |  |  |
| **Frontal β** | GD | 30.42 ± 10.49 | 0.83 | 0.407 | 0.19 |
|  | HC | 32.68 ± 13.46 |  |  |  |
| **Temporal Δ (L)** | GD | 43.53 ± 16.79 | –1.60 | 0.115 | –0.36 |
|  | HC | 37.96 ± 12.62 |  |  |  |
| **Temporal θ (L)** | GD | 26.22 ± 6.70 | –0.50 | 0.621 | –0.11 |
|  | HC | 25.21 ± 11.43 |  |  |  |
| **Temporal α (L)** | GD | 42.56 ± 19.69 | 0.02 | 0.983 | 0.01 |
|  | HC | 42.66 ± 20.23 |  |  |  |
| **Temporal β (L)** | GD | 27.44 ± 8.72 | 1.57 | 0.119 | 0.36 |
|  | HC | 31.59 ± 14.71 |  |  |  |
| **Temporal Δ (R)** | GD | 41.96 ± 15.95 | –0.48 | 0.635 | –0.11 |
|  | HC | 40.28 ± 14.35 |  |  |  |
| **Temporal θ (R)** | GD | 24.17 ± 7.62 | –0.34 | 0.737 | –0.08 |
|  | HC | 23.39 ± 12.91 |  |  |  |
| **Temporal α (R)** | GD | 45.46 ± 21.86 | –0.60 | 0.549 | –0.14 |
|  | HC | 42.44 ± 21.79 |  |  |  |
| **Temporal β (R)** | GD | 26.47 ± 9.85 | 1.30 | 0.197 | 0.30 |
|  | HC | 30.09 ± 14.86 |  |  |  |
| **Parietal Δ (L)** | GD | 23.75 ± 12.88 | –1.23 | 0.224 | –0.28 |
|  | HC | 20.47 ± 9.58 |  |  |  |
| **Parietal θ (L)** | GD | 15.53 ± 4.61 | –0.12 | 0.906 | –0.03 |
|  | HC | 15.33 ± 10.03 |  |  |  |
| **Parietal α (L)** | GD | 41.60 ± 20.46 | –0.03 | 0.975 | –0.01 |
|  | HC | 41.45 ± 21.52 |  |  |  |
| **Parietal β (L)** | GD | 15.89 ± 7.12 | 1.59 | 0.116 | 0.36 |
|  | HC | 19.71 ± 14.03 |  |  |  |
| **Parietal Δ (R)** | GD | 23.03 ± 12.63 | –0.77 | 0.441 | –0.18 |
|  | HC | 20.94 ± 10.41 |  |  |  |
| **Parietal θ (R)** | GD | 15.06 ± 4.83 | –0.01 | 0.994 | 0.00 |
|  | HC | 15.05 ± 10.68 |  |  |  |
| **Parietal α (R)** | GD | 42.61 ± 20.77 | –0.09 | 0.926 | –0.02 |
|  | HC | 42.15 ± 22.21 |  |  |  |
| **Parietal β (R)** | GD | 15.83 ± 7.70 | 1.19 | 0.237 | 0.27 |
|  | HC | 18.77 ± 14.13 |  |  |  |
| **Occipital Δ (L)** | GD | 20.17 ± 11.73 | –0.78 | 0.435 | –0.18 |
|  | HC | 18.23 ± 9.36 |  |  |  |
| **Occipital θ (L)** | GD | 14.44 ± 5.42 | –0.59 | 0.559 | –0.13 |
|  | HC | 13.54 ± 8.28 |  |  |  |
| **Occipital α (L)** | GD | 44.34 ± 20.17 | –0.02 | 0.981 | –0.01 |
|  | HC | 44.23 ± 21.20 |  |  |  |
| **Occipital β (L)** | GD | 16.76 ± 7.50 | 1.34 | 0.183 | 0.31 |
|  | HC | 20.13 ± 14.65 |  |  |  |
| **Occipital Δ (R)** | GD | 20.51 ± 12.79 | –0.95 | 0.343 | –0.22 |
|  | HC | 18.00 ± 9.32 |  |  |  |
| **Occipital θ (R)** | GD | 13.79 ± 5.36 | –0.63 | 0.532 | –0.14 |
|  | HC | 12.76 ± 9.13 |  |  |  |
| **Occipital α (R)** | GD | 45.41 ± 21.13 | 0.04 | 0.966 | 0.01 |
|  | HC | 45.62 ± 21.18 |  |  |  |
| **Occipital β (R)** | GD | 15.93 ± 8.39 | 1.53 | 0.130 | 0.35 |
|  | HC | 19.96 ± 14.91 |  |  |  |

**Note.** Values represent mean ± SD relative EEG power (%) in each frequency band and ROI for GD and HC groups. Negative *t* values reflect higher mean power in GD. *p* < .05 is considered significant (none reached significance; left-temporal delta and beta showed trend-level differences).
