# Supplementary material for: Resting-state EEG power and machine-learning classification in adult males with gambling disorder
Source: Front Hum Neurosci. 2026 Jan 13;19:1725528. doi: 10.3389/fnhum.2025.1725528 (PMC12835224; doi:10.3389/fnhum.2025.1725528)
Supplement: Supplementary file 3 [file Table_3.docx]

S3. Correlations between Absolute EEG Power and South Oaks Gambling Screen (SOGS) Scores after Benjamini–Hochberg FDR Correction

| **Region / Band** | ***r*** | ***p*** | ***p*FDR** | **Significance** |
| --- | --- | --- | --- | --- |
| Right Parietal β | .497 | < .001 | .008 | **Significant** |
| Right Frontal β | .399 | .005 | .019 | **Significant** |
| Left Frontal β | .339 | .020 | .054 | ns |
| Left Temporal β | .381 | .008 | .028 | **Significant** |
| Right Temporal β | .446 | .002 | .014 | **Significant** |
| Left Occipital β | .403 | .005 | .027 | **Significant** |
| Right Occipital β | .404 | .005 | .027 | **Significant** |
| Right Temporal α | .325 | .026 | .071 | ns |
| Left Occipital α | .327 | .025 | .074 | ns |
| Right Parietal δ | .383 | .008 | .055 | ns |
| Right Parietal θ | .354 | .015 | .067 | ns |
| Left Frontal θ | .308 | .035 | .095 | ns |
| Left Frontal δ | .290 | .048 | .103 | ns |

*Note.* *ns* = not significant after FDR correction. Reported values are Pearson’s *r*, two-tailed *p*, and Benjamini–Hochberg adjusted *p*FDR. Only the strongest β-band associations remained significant following correction.

Following reviewer recommendations, we applied the Benjamini–Hochberg False Discovery Rate (FDR) correction (Benjamini & Hochberg, 1995) to control for multiple comparisons among EEG–SOGS correlations. After adjustment, the majority of strong β-band correlations (right parietal, bilateral occipital, right temporal, and right frontal) remained significant, whereas weaker δ, θ, and α correlations did not survive correction. These results confirm that the association between gambling severity and resting β-band power is statistically robust and unlikely to reflect Type I error.
